# 

**Published:** 1838-01

**Authors:** 


### QUARTERLY JOURNAL

OF

### PRACTICAL MEDICINE AND SURGERY

LIBRARY OF THE

Orleans Parish Medical Society

EDITED BY

JOHN FORBES M.D. F.R.S.

AND

JOHN CONOLLY M.D.

EDITORS OF THE CYCLOPÆDIA OF PRACTICAL MEDICINE

---

VOL. V.

JANUARY—APRIL 1838

LONDON
JOHN CHURCHILL PRINCES STREET SOHO
MDCCCXXXVIII

LONDON:

PRINTED BY C ADLARD, BARTHOLOMEW CLOSE.

### BOOKS RECEIVED FOR REVIEW.

ENGLISH.

1. The Philosophy of Health; or, an Exposition of the Physical and Mental Constitution of Man, with a View to the Promotion of Human Longevity and Happiness. By Southwood Smith, M.D. &c. Vol. II.—London, 1837. 12mo. pp. 448. 7s.

2. Guy's Hospital Reports. No. V. October, 1837. Edited by G. H. Barlow, M.A. L.M. &c., and J. P. Babington, M.A. &c.—London. 8vo. pp. 240. 6s.

3. Elements of Physiology. By J. Müller, M.D., Professor of Anatomy and Physiology in the University of Berlin. Translated from the German, with Notes, by William Baly, M.R.C.S. &c. Illustrated with steel Plates and wood Engravings. Part I.—London, 1837. 8vo. pp. 428. 9s.

4. A concise Treatise on Operative Surgery, describing the Methods adopted by the English, Continental, and American Surgeons; selected for the use of junior Practitioners and Students. Illustrated by Plates. By W. P. Cocks, Surgeon.—London, 1837. 8vo. pp. 375. 14s.

5. Elements of Anatomy. By Jones Quain, M.D. Fourth Edition, revised and enlarged. Illustrated with steel Plates and Woodcuts. — London, 1837. 8vo. Part I. pp. 388, 12. 22s.

6. Elements of Chemistry; including the recent Discoveries and Doctrines of the Science. By the late Edward Turner, M.D. Sixth Edition; enlarged and revised by Professor Liebeg and Wilton G. Turner. Part I.—London, 1837. 8vo. pp. 410. 7s.

7. A Discourse on some of the Diseases of the Knee-joint; delivered before the Massachuset's Medical Society, at their annual Meeting, May 31, 1837. By G. Hayward, M.D., Professor of Surgery in Harvard University.—Boston, 1837. 8vo. pp. 28.

8. Memoranda on difficult Subjects in Anatomy and Surgery; being a Pocket Companion for Students preparing for the College of Surgeons. By Robert Druit, M.R.C.S.—London, 1837. 48mo. 2s.

9. Remarks on the Memorial of the Town-Council of Edinburgh, respecting the Professorship of Medicine and general Pathology. By the present Professor.—8vo. pp. 43.

10. Report of the Proceedings at the first annual Meeting of the Southern District Branch of the Provincial Medical and Surgical Association, held at Winchester, June 5, 1837.—8vo. pp. 30.

11. Transactions of the Medical Society of the State of New York. Vol. I. II. III. —Albany, 1832-1837. 8vo. pp. 372, 388, 362.

12. Hints to Mothers for the Management of Health during the Period of Pregnancy, and in the Lying-in Room; with an Exposure of the popular Errors in connexion with those Subjects. By Thomas Bull, M.D., Physician-Accoucheur to the Finsbury Midwifery Institution, &c.—London, 1837. 12mo. pp. 174. 5s.

13. On the Use of Auscultation and Percussion in the Diagnosis of Diseases of the Organs of Respiration and Circulation; with Directions for the Employment of Inspection, Succussion, Palpation, and Mensuration of the Thorax. By Julius Wolff, M.D.—London, 1837. 8vo. pp. 200. 6s.

14. A Conspectus of the Pharmacopœias of the London, Edinburgh, and Dublin Colleges of Physicians; being a Practical Compendium of Materia Medica and Pharmacy. By A. T. Thomson, M.D. F.L.S. &c. Tenth Edition.— London, 1837. 12mo. pp. 190. 5s. 6d.

15. Cases of Hidrosis, or Hidrotic Fever; with Remarks. By J. C. W. Lever, M.R.C.S.—London, 1837. 8vo. pp. 24.

16. Outlines of Comparative Anatomy. By R. E. Grant, M.D. &c. Part IV., containing Digestive Organs, Chyliferous and Sanguiferous Systems.—London, 1837. 8vo. pp. 80. 4s.

17. Practical Surgery; with 120 Engravings on Wood. By Robert Liston, Surgeon.—London, 1837. 8vo. pp. 494. 22s.

18. The Works of John Hunter. Edited by Mr. Palmer. Vol. III. 8vo. 17s. 6d.

19. Treatise on Hernia: comprising the Surgical Anatomy, Operative Surgery, and Treatment of that important Disease in all its Forms; as also a newly proposed Operation for the Relief of strangulated Hernia. By M. W. Hilles.—London, 1838. 12mo. pp. 52. 2s.

20. First Principles of Surgery; being an Outline of Inflammation and its Effects. By G. T. Morgan, A.M., Lecturer on Surgery in Aberdeen. Part II.—8vo. pp. 207. 5s.

21. Prize Thesis, on the Presence of Air in the Organs of Circulation. By J. R. Cormack, M.D.—Edinburgh, 1837. 8vo. pp. 56. 1s. 6d.

22. Essay on the Classification of the Insane. By M. Allen, M.D. — London, (no date.) 8vo. pp. 212; with Plates. 6s.

23. Changes produced in the Nervous System by Civilization, considered according to the Evidence of Physiology and the

Philosophy of History. By Robert Verity, M.D.—London, 1837. 8vo. pp. 79. 5s. 6d.

24. Obstetric Plates, with Explanations, selected from the Anatomical Tables of William Smellie, M.D. (Republished.)—London, 1837. 8vo. pp. 32; Plates, 12. 5s.

25. Institutes of Surgery: arranged in the order of the Lectures delivered in the University of Edinburgh. By Sir Charles Bell, K.H.G. F.R.SS. L. and E., Professor of Surgery in the University of Edinburgh. In two vols. Vol. I.—Edinburgh, 1838. 8vo. pp. 352. 7s. 6d.

26. A Table, exhibiting the Results of Observations by the Thermometer, Barometer, Hygrometer, and Rain-gauge, &c., from 1829 to 1836, inclusive, at Exeter. By T. F. Barham, M.B.—One sheet, folio. Exeter, 1837.

27. A Letter to the Inhabitants of Ceylon on the Advantages of Vaccination. By J. Kinnis, M.D.—Ceylon, 1837. 8vo. pp. 28.

28. The Question concerning the Sensibility, Intelligence, and instinctive Actions of Insects. By David Barham, M.D., one of the Radcliffe Travelling Fellows, &c.—Paris, 1837. 8vo. pp. 54.

29. On the Diseases of the Rectum. By James Syme, F.R.S.E., Professor of Clinical Surgery in the University of Edinburgh.—Edinburgh, 1838. 8vo. pp. 138. 5s.

30. Remarks on the Treatment of Fractures of the Extremities without the Aid of Splints: with Cases. By J. F. Burke, M.R.C.S.—London. 1837. 8vo. pp. 39. 2s.

31. The British Medical Almanack, 1838; with a Supplement. Edited by William Farr.—London, 1837. 12mo. pp. 224. 3s.

32. Powers of the Roots of the Nerves in Health and in Disease. Likewise, on Magnetic Sleep. By Herbert Mayo, esq. F.R.S.—London, 1837. 8vo. pp. 39.

33. A Lecture on the Nature and Cultivation of the Medical Profession; intended as a Guide to Students. By G. T. Morgan, A.M., Lecturer on Surgery in Aberdeen.—London, 1838. 8vo. pp. 28. 1s.

34. Practical Remarks on the Diseases of the Skin, on the external Signs of Disorder, and on the Constitutional Peculiarities during Infancy and Childhood. By W. C. Dendy, Surgeon to the Infirmary for Children, &c.—London, 1837. 8vo. pp. 153. 6s. 6d.

FOREIGN.

1. Rede zur Feier des 43sten Stiftungstages des Königlichen Med. Chir. Fred. Wilh. Instituts, am 2ten August, 1837, gehalten. Von Dr. J. F. K. Hecker, Königl. Professor der Heilkunde.—Berlin, 1837. 8vo. pp. 19.

2. Ueber Virilescenz und Rejuvenescenz thierisher Körper: Ein Beitrag zur Lehre von der regelwidrigen Metamorphose Organischer Körper. Von Dr. C. W. Mehliss praktischem Artze in Liebenwerda.—Leipzig, 1838. 8vo. pp. 114.

3. Lehrbuch der Geburtshülfe zum Unterricht für Hebammen. Von Dr. J. C. Stark, &c.—Jena, 1837. 8vo. pp. 292.

4. Die freie Hansestadt Bremen und ihr Gebiet in topographischer, medizinischer, und naturhistorischer Hinsicht geschildert, von Ph. Heineken, M.D. &c.—Bremen, 1837. 8vo. pp. 215.

5. Arsberättelse om Svenska Läkare-Sällskapets Arbeten. (Eight Volumes.)—Stockholm, 1825–1832. 8vo.

6. Acta Solennia quibus tertium Jubilæum sacrorum in regno Daniæ reformatorum, Die xxxi. Oct. An. 1836, in Aula Academica celebravit Universitas Regia Hauniensis.—Hauniæ, 1837. Fol. pp. 84.

7. Observationes aliquot ad Physiologiam Gangli Ophthalmici pertinentes. Dissertatio inauguralis quam conscripsit Henricus Wendt, Hafniensis.—Kiliæ, 1834. 4to. pp. 22.

8. Die Influenza oder Grippe, nach den Quellen historisch-pathologisch dargestellt. Eine gekrönte Preisschrift. Von Dr. Gottlieb Gluge.—Minden, 1837. 8vo. pp. 167.

9. Ueber das Seebaden und das Norderneyer Seebad. Von Dr. Carl Mühry, Lehrer der Physiologie, &c. zu Hannover.—Hannover, 1836. 8vo. pp. 184.

10. Darstellungen und Ansichten zur Vergleichung der Medicin in Frankreich, England, und Deutschland. Von Dr. C. Mühry.—Ib. 1836. Pp. 283.

11. Der Artzt im Menschen, oder die Heilkraft der Natur. Von Dr. G. F. Ch. Greiner, Hofmedicus und Stadtphysicus zu Altenburg.—Altenburg, 1827, 1829. Two Vols. 8vo. pp. 484, 487.

12. Die Vergleichende Osteologie des Schläfenbeins. Zur Vereinfachung der herschenden ansichten bearbeitet von Eduard Hallmann. Mit iv. Kupfertafeln.—Hannover, 1837. 4to. pp. 130.

13. Zur Vermittelung der Extreme in der Heilkunde. Von Th. Stürmer, M.D. &c.—Leipzig, 1837. 8vo. pp. 448.

14. Anthropologischer Beitrag zur Erfahrung der psychischen Krankheit, &c. C. P. Möller, M.D. &c. — Mainz, 1837. 8vo. pp. 507.

15. Physiologisch-Therapeutische Untersuchungen über das Veratrin. Von Dr. Fred. A. Forcke.—Hannover, 1837. 8vo. 146.

16. Periodologie oder die Lehre von den periodischen Veränderungen im Leben des gesunden und kranken Menschen. Von Dr. A. M. Baumgarten Crusius.—Halle, 1836. 8vo. pp. 457.

